# Study on Road Performance of Cement Fly Ash Stabilized Steel Slag—Concrete Recycled Macadam

**DOI:** 10.3390/ma14247530

**Published:** 2021-12-08

**Authors:** Hongbo Li, Yufei Tong, Hubiao Zhang, Xuanshuo Zhang, Junku Duan

**Affiliations:** 1 College of Civil and Hydraulic Engineering, Ningxia University, Yinchuan 750021, China; tongyufei1028@163.com (Y.T.); zhanghubiao1222@163.com (H.Z.); zxsnikea@163.com (X.Z.); 2 Ningxia Research Center of Technology on Water-Saving Irrigation and Water Resources Regulation, Yinchuan 750021, China; 3 Ningxia Center of Research on Earthquake Protection and Disaster Mitigation in Civil Engineering, Yinchuan 750021, China; 4 Ningxia Huasheng Energy Saving and Environmental Protection Technology Co., Ltd., Yinchuan 750021, China; duanjunku2021@163.com

**Keywords:** unconfined compressive strength, indirect tensile strength, relative dynamic elastic modulus, formation mechanism of strength, strength attenuation model

## Abstract

In order to promote the application of steel slag in road engineering, improve its utilization rate and solve the environmental problems caused by its large accumulation, unconfined compressive strength (UCS) test, indirect tensile strength (ITS) test, freeze-thaw cycle test, dry shrinkage and temperature shrinkage test tests with different steel slag contents were carried out. And the strength formation mechanism of steel slag in base material was revealed by SEM. The results show that the strength of the mixture initially increased and then decreased with increasing steel slag content. The frost resistance increased with increasing steel slag content, which should be limited to no more than 75%. Increasing the steel slag content improved the drying shrinkage resistance but was not conducive to the temperature shrinkage resistance. Microscopic analysis shows that adding a suitable amount of steel slag generated a gel material that was distributed inside the pores. This increased the density of the hardened slurry structure, which improved the strength. The research can provide scientific basis for the application and promotion of steel slag in road base.

## 1. Introduction

Semirigid bases are the main form of road bases in China, and they offer the advantages of a high bearing capacity, moderate stiffness, and good slab properties. In recent years, the rapid development of China’s highway engineering industry has led to serious overexploitation of natural gravel, which has caused irreversible environmental damage. Thanks to the government’s increasing emphasis on protecting the environment, China has explicitly banned the exploitation of natural gravel, which has resulted in shortages of stone for road construction and price increases. As a solid waste of coal, fly ash has been widely used because of its large output and low cost. Khan and Ahmad [1,2,3,4] used advanced machine learning technology to predict the strength of fly ash concrete, which accelerated the utilization of fly ash in construction industry.

Steel slag (SS) is an industrial waste product of the steelmaking process and accounts for 15–20% of the crude steel output [5]. The amount of SS produced has increased by millions of tons each year, but the utilization rate is less than 20% in China [6]. The accumulation of SS not only occupies land resources but also increases environmental pollution and destruction [7,8]. Studies [9,10,11,12,13] have shown that SS is a good building material for roads owing to its hard texture and excellent wear resistance. Thus, many researchers have focused on using SS as an aggregate instead of gravel in road bases. Developed countries such as Germany and the United States use more than 50% of SS in road engineering construction. In Germany, SS has been applied to the surface and base of high-grade highways. The United States has developed a comprehensive system for applying SS to highways. Chand [14] showed that SS has wear and compressive properties close to those of basalt, and it has a density and water absorption greater than those of natural aggregates. Marina [15] analyzed the technological and environmental implications of using SS to replace natural aggregate in pavement, which would potentially reduce carbon emissions by more than 14%. Kim [16] used SS instead of natural aggregate and added amorphous metallic fiber to improve the performance of SS in a mortar.

Many scholars in China have researched applying SS to semirigid bases. Zhou [17] discussed the effect of adding 30–60% cement fly ash on the mechanical properties and expansion behavior of SS. The results showed that fly ash inhibits the expansion of SS and that the resulting mixture has good mechanical properties and volume stability. Wang [18,19,20] studied the hydration and cementitious properties of SS. The results showed that SS has a slow hydration rate; the inert component cannot fill the pores of the slurry, while the cementitious component improves the hardening effect of the slurry. Liang [21] used cement-stabilized SS as a base and paved a test section of an expressway. The results showed that replacing gravel with SS resulted in a 7-d unconfined compressive strength (UCS) of greater than 9 MPa as well as good elastic deformation resistance. Huang [22] evaluated the mechanical properties, volume stability, and environmental impact of mixtures with different cement and SS contents through a series of indoor tests. The results showed that a mixture with 50% SS content had the best overall mechanical properties and good application prospects. Xiao [23] used a self-made composite activator to realize activated steel slag powder (ASSP) and evaluated the compressive and splitting resilience modulus, frost resistance, drying shrinkage, and temperature shrinkage as well as microstructure. The results showed that using a large amount of ASSP in cement-stabilized macadam as a pavement base is feasible. Ji [24] showed that adding SS to the base material significantly reduced the erosion mass loss and increased the UCS and splitting strength. Based on the results, a mixture of 65% SS was recommended.

It can be seen from the research summary that scholars around the world have done a lot of research on the physical and chemical properties of steel slag and the mechanical properties after replacing crushed stone aggregate. They have considered the influence of origin and steelmaking process on the basic properties of steel slag, combined with the specific application environment, put forward specific construction suggestions, and achieved certain research results. However, the related tests are still mainly based on mechanical properties. As an environmentally friendly road building material, the influence of steel slag on the mixture is complex and long-term. Durability and crack resistance are also important, and the related research is relatively small, which needs further discussion.

**Figure 1:**
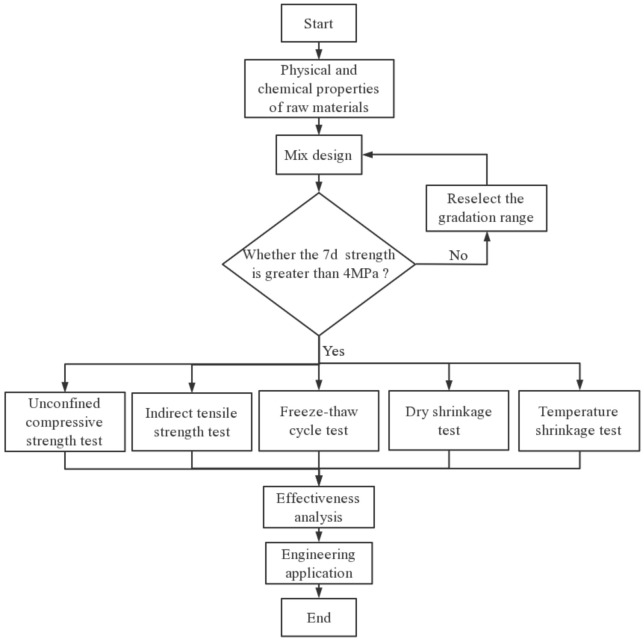
Research flow diagram.

**Figure 2:**
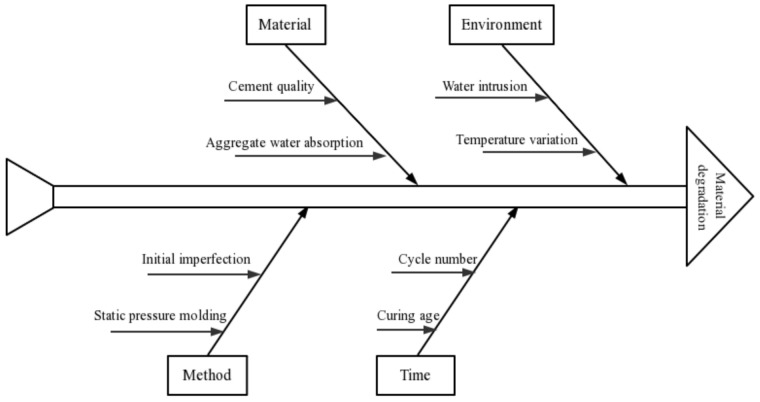
Ishikawa diagram that contains the cause of material degradation during freezing and thawing.

In this study, SS and concrete-recycled macadam (CRM) were mixed together and stabilized with cement and fly ash. Five mixtures were then designed to evaluate the effects of the SS content on the mechanical properties, durability, and shrinkage properties. Figure 1 shows the flowchart of this article, and Figure 2 is the Ishikawa diagram that contains the cause of material degradation during freezing and thawing. This study may help guide the application of SS to semirigid bases to realize the efficient recycling of industrial solid waste and address the shortage of natural gravel.

## 2. Materials and Methods

### 2.1. Materials

Ordinary Portland cement PO 42.5 with a density of 3.16 g/cm^3^ was used. Grade III fly ash with a density of 2.51 g/cm^3^ was acquired from Ningxia Yinchuan Xixia District Thermal Power Plant (Yinchuan, China). Aged and hot stuffy SS was obtained from a steel plant in Shizuishan City, Ningxia, China. The materials were processed by multistage crushing and washing in a drum and water. CRM was obtained in the form of crushed concrete from a construction waste induction point in Yinchuan City. After secondary crushing by a jaw crusher, the CRM was divided into four grades. Table 1 presents the chemical compositions of the cement, fly ash, and SS. Table 2 presents the technical indices of the cement. Table 3 presents the physical properties of SS and CRM, which were used as performance indices.

**Figure 3:**
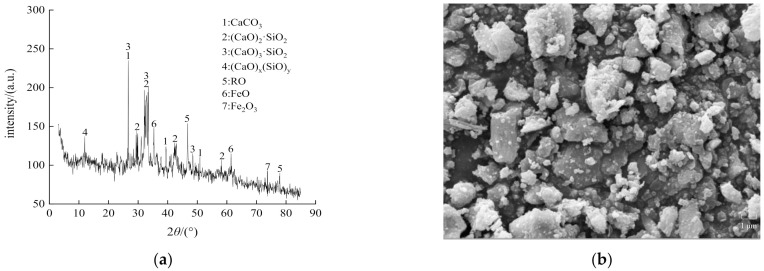
(**a**) Mineral composition and (**b**) microstructure of steel slag.

**Figure 4:**
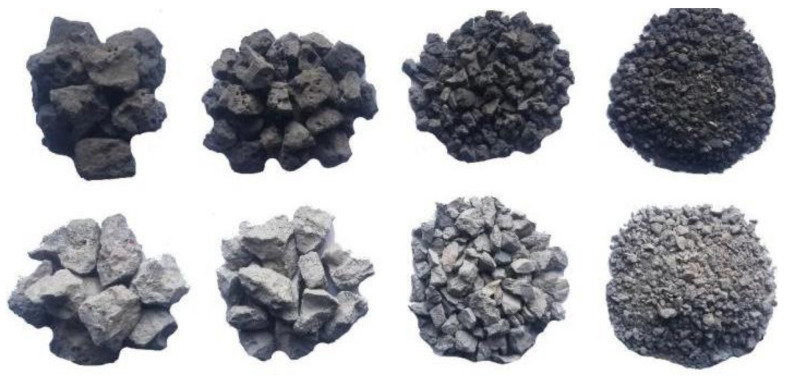
Steel slag and concrete-recycled macadam with different particle sizes.

Figure 3 shows the mineral composition and microstructure of the SS obtained by X-ray diffraction and scanning electron microscopy (SEM), respectively [25,26]. The main mineral components included CaCO_3_, C_2_S, C_3_S, and the RO phase (continuous solid solution composed of divalent metal oxides). The SEM images showed that the SS had a plate-like crystal structure, and its surface was rough and uneven with many small pores and small particles [27]. Figure 4 shows SS and CRM with different particle sizes. The rich porous structure on the surface of steel slag increases the wetting contact surface between cement mortar and steel slag, enhances the interfacial adhesion strength and skeleton embedded force of steel slag mixture, and helps to greatly improve the road performance of cement fly ash stabilized steel slag-concrete macadam mixture. The cement mortar on the surface of recycled aggregate concrete is rough and porous, and it is easy to absorb water. Its strength is lower than that of ordinary gravel, and it is easier to crush. Its crushing index is high, but it meets the requirements of road base materials.

### 2.2. Mix Design

Mixtures were designed according to CF-A-2 in YB/T 4184-2018 [28]. The cement and fly ash contents were fixed to 3% and 12%, respectively. The composite of SS and CRM was used as the aggregate, and the maximum nominal particle size was no more than 26.5 mm. Aggregates were screened to realize mixtures with 0%, 25%, 50%, 75%, and 100% SS. Table 4 presents the designed mixtures and the gradations of their particle sizes.

### 2.3. Compaction Test

Heavy compaction tests were performed according to the method given in T0804-1994 of JTG E51-2009 [29] to determine the maximum dry density and optimum moisture content of the mixtures according to the SS content.

### 2.4. Immersion Expansion Rate Test

The immersion expansion rate test method in GB/T 24175-2009 [30] was used to compact specimens of the different mixtures. Three specimens of each mixture were tested for 15 specimens in total. Each specimen was placed in a constant-temperature water bath and immersed to a depth of more than 2.5 cm. The specimens were then heated to a water bath temperature of 90 °C for 6 h each day over 10 days. The readings of the dial indicator were read before heating each day. The immersion expansion rate of the specimens was then measured to evaluate the water stability of the mixtures according to the SS content.

### 2.5. Unconfined Compressive Strength Test

Specimens with dimensions of 150 mm × Φ150 mm were prepared by the static pressure method for a compaction degree of 98% and were then wrapped with plastic bags. The specimens were cured in a standard maintenance room with a temperature of 20 °C ± 2 °C and humidity of >95% for 7, 28, 56, and 90 days. On the last day of the maintenance period, the specimens were soaked in water for 24 h, and the surface moisture was wiped off. Then, the UCS of the specimens at different ages was tested on a test machine with a loading speed of 1 mm/min.

### 2.6. Indirect Tensile Strength Test

The test method of T0806-1994 in JTG E51-2009 [29] was used to test the indirect tensile strength (ITS) of the specimens. Specimens were prepared and cured in the same manner as for the UCS test.

### 2.7. Freeze–Thaw Cycle Test

The test method of T0858-2009 in JTG E51-2009 [29] was used to perform the freeze–thaw cycle test. Specimens were formed with dimensions of 150 mm × Φ150 mm and were cured in the maintenance room for 28 days. On day 27, the specimens were soaked in water for 24 h. After saturation, the water on the surfaces was dried off, and the specimens were weighed to determine the reference mass. The uniaxial compressive strength test was carried out to determine the reference strength of each specimen. For the freeze–thaw cycle, specimens were then placed in a constant-temperature testing machine to freeze at −18 °C for 16 h. Then, they were placed in a constant-temperature water tank to thaw at 20 °C for 8 h. After thawing, each specimen was taken out of the tank for 15 min. The water on the surfaces was wiped off, and the specimens were weighed. The average value was taken to determine the UCS after 5, 10, 15, 20, 25, 30, 35, and 40 freeze–thaw cycles. The frost resistance was evaluated in terms of the mass change and strength loss. The NM-4A nonmetallic ultrasonic testing analyzer produced by Beijing Kangkerui Company (Beijing, China) was used to detect internal defects of specimens and establish a strength attenuation model.

### 2.8. Dry Shrinkage Test

The dry shrinkage test was performed according to the method in T0854-2009 in JTG E51-2009 [29]. Specimens were formed with dimension of 150 mm × Φ150 mm and cured in the maintenance room for 7 days. On day 6, the specimens were soaked in water for 24 h. Then, the water on the surfaces of the saturated specimens was dried off, and the specimen surfaces were smoothed and weighed by using an angular grinder. The specimen surface was then flattened with epoxy resin, upon which a glass sheet was pasted. Finally, the specimens were placed in a drying chamber. The dial gauge was fixed, and the initial reading was recorded. Six specimens were prepared for each mixture: three were used to determine the dry shrinkage according to the water loss rate, and the other three were used to determine the shrinkage deformation. The average of the measurement results was taken. Data were recorded daily for the first 7 days, every 2 days after 7 days, and at 60 and 90 days after 29 days.

### 2.9. Temperature Shrinkage Test

The temperature shrinkage test was carried out according to the method in T0855-2009 in JTG E51-2009 [29]. Specimens were formed with dimensions of 150 mm × Φ150 mm and were cured in the maintenance room for 7 days. On day 6, the specimens were taken out and soaked in water for 24 h. They were then dried in an oven at 105 °C for 12 h. The dried specimens were placed in a drying chamber and were then cooled to room temperature. The center of the specimen surface was ground with sandpaper. Then, a strain gauge with temperature compensation was pasted there, and the lead was connected to the interface of the static strain gauge. The test started from a high temperature. The temperature range was set to 60 °C to −20 °C. The stages of the test had temperature differences of 10 °C. The cooling rate was 1 °C/min, and the temperature was kept at each stage for 4 h. Parallel tests were carried out on three specimens of each mixture, and the measurement results were averaged to determine the effect off the SS content.

## 3. Results and Discussion

### 3.1. Compaction Test Results

**Figure 5:**
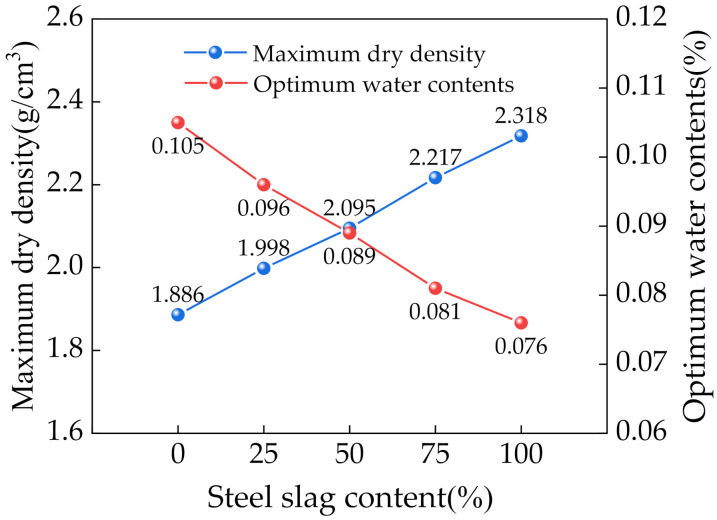
Variation curves of the maximum dry density and optimum moisture content.

Figure 5 shows the maximum dry density and optimum moisture content of the mixtures with different SS contents. Increasing the SS content gradually increased the maximum dry density and decreased the optimum moisture content. This was mainly because SS was denser than CRM and had a lower water absorption rate.

### 3.2. Immersion Expansion Rate Test Results

**Figure 6:**
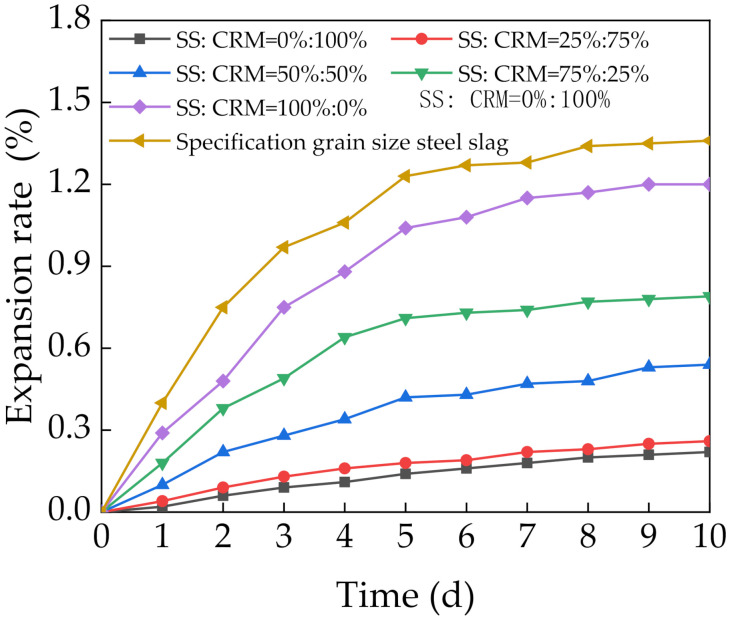
Variation curve of the immersion expansion rate.

Figure 6 shows the immersion expansion rates of the mixtures. The immersion expansion rate initially increased rapidly and then increased slowly at later stages. The final immersion expansion rate was less than 2%, which meets the requirements of GB/T 24175-2009 [30]. Increasing the SS content of the mixtures gradually increased the immersion expansion rate. The immersion expansion rate was 0.26% at 25% SS, which was almost identical to that at 0% SS. Thus, SS had little effect on the immersion expansion rate when the SS content was low. Only higher SS contents had an effect on the immersion expansion rate. Specimens with 50%, 75%, and 100% SS had immersion expansion rates that were respectively 2.45, 3.59, and 5.45 times that of the specimens without SS. However, the immersion expansion rates were still less than that of SS graded according to standard particle sizes. This was attributed to the following. First, SS is a finer aggregate than CRM, so increasing its content increases the specific surface area. This makes the active material chemical reaction faster, which increases the immersion expansion rate. Second, the cement and fly ash in the mixtures played a stabilizing role and caused some shrinkage, which inhibited the expansion of SS.

### 3.3. Unconfined Compressive Strength Test Results

**Figure 7:**
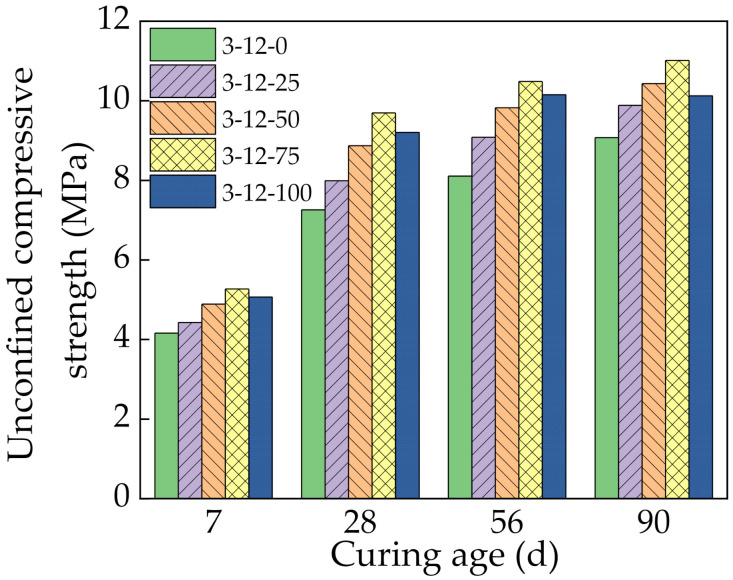
Unconfined compressive strength with different steel slag contents.

Figure 7 shows the UCS results of the mixtures at 7, 28, 56, and 90 days. The UCS increased with the curing age, and it initially increased and then decreased with increasing SS content. At 7 days, the mixture without SS had a UCS of 4.2 MPa. Increasing the SS content to 25%, 50%, 75%, and 100% increased the UCS by 6.4%, 17.5%, 26.7%, and 21.7%, respectively. This can be attributed to three main reasons. First, the SS had a smaller crushing value and rougher surface than CRM. It also had high hardness and crushing resistance, which are helpful to the strength formation. Thus, increasing the SS content increased the strength of the mixture. Second, the SS had active components such as C_3_S, C_2_S, C_3_A, and C_4_AF. These minerals react with water to produce hydration products during the cementation process, which increases the strength of the mixture. Third, the SS contained a small amount of free CaO, and its hydration in water resulted in volume expansion. This fills the pores of the mixture, which increases the density and thus improves the strength of the mixture [31].

The UCS increased only 6.4% at 25% SS. This is because SS has little effect on the strength of the mixture at low contents and cannot play a dominant role. The UCS of 100% SS was lower than 75% SS, which indicates that the SS content can be optimized. When the SS content was too high, the high porosity of SS increased the porosity of the specimen. Some cement was adsorbed and wrapped inside the surface pores of SS, which isolated water molecules and made them unavailable for hydration, which is not conducive to strength formation. In addition, the SS had a relatively high content of active substances, which resulted in excessive volume expansion after their reaction. The original pores of the mixture were not sufficient to absorb this volume expansion, which increased the internal stress. When the cumulative internal stress accumulation reached a certain level, some small cracks were generated in the mixture, which reduced the strength [32].

From 7 days to 28 days, the UCS of the specimens with 0%, 25%, 50%, 75%, and 100% SS content clearly increased by 74.4%, 80.5%, 81.5%, 83.9%, and 81.7%, respectively. This was mainly because SS promoted the reaction of cement. The SS also contained C_2_S, C_3_S, and other minerals with hydration activity. Similar to cement clinker, the hydration of these minerals continuously generated cementitious products, which rapidly increased the strength of the mixture. At the same time, the active substances Al_2_O_3_, SiO_2_, and Ca(OH)_2_ in the SS and fly ash resulted in secondary hydration reactions that generated various gels such as C–A–H, C–S–H, and AFt. The chemical reactions are given below:

(1) $$\mathrm{Ca}{\left(\mathrm{OH}\right)}_{2}+{\mathrm{SiO}}_{2}+2H_{2}O\to \mathrm{CaO}\cdot {\mathrm{SiO}}_{2}\cdot 2H_{2}O\;\mathrm{Ca}{\left(\mathrm{OH}\right)}_{2}+{\mathrm{Al}}_{2}O_{3}+2H_{2}O\to \mathrm{CaO}\cdot {\mathrm{Al}}_{2}O_{3}\cdot 2H_{2}O\;3\mathrm{CaO}\cdot {\mathrm{Al}}_{2}O_{3}+\left({\mathrm{CaSO}}_{4}\cdot H_{2}O\right)+H_{2}O\to 3\mathrm{CaO}\cdot {\mathrm{Al}}_{2}O_{3}\cdot {\mathrm{CaSO}}_{4}\cdot 2H_{2}O$$

The formation of these gels bound the interface between the SS, CRM, and cement mortar more closely together. This significantly improved the compactness and cementation strength inside the specimen and directly improved the UCS.

At 56 and 90 days, the growth rate of the UCS decreased significantly. This is because further progression of the secondary hydration reaction decreased the active substances such as Ca(OH)_2_ available in the specimens, which slowed down the reaction rate. At the same time, the water–binder ratio of the specimens decreased, and insufficient water was available for hydration, which reduced the growth rate of the strength at later stages.

### 3.4. Indirect Tensile Strength Test Results

**Figure 8:**
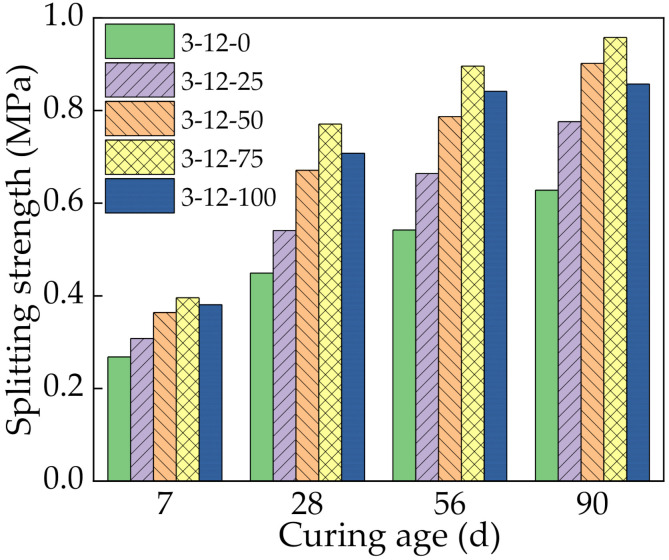
Indirect tensile strength with different steel slag contents.

Figure 8 shows the ITS results of the mixtures with different SS contents at 7, 28, 56, and 90 days. The ITS increased with the curing age, and it initially increased and then decreased with increasing SS content, similar to the UCS. At 7 days, the variation law and mechanism of the ITS were the same as for the UCS. From 7 days to 28 days, the ITS of specimens with 0%, 25%, 50%, 75%, and 100% SS rapidly increased by 67.5%, 75.7%, 84.3%, 94.7%, and 85.8%, respectively. The key factors that affected the ITS were the hydration reaction of the binder and the bonding effect between the binder and aggregate [33]. During this time, the main reaction was the hydration of the cement and SS. These formed hydration products that fully bonded the loose aggregate to form a dense structural layer, which greatly improved the ITS [34]. Meanwhile, with the extension of curing age, the active substances in slag and fly ash gradually undergo secondary hydration, which consumed Ca(OH)_2_, inhibited the growth of Ca(OH)_2_ grains, and reduced the thickness of the interfacial transition zone. The continuous consumption of Ca(OH)_2_ generated gels that further improved the ITS of the specimen. At 56 and 90 days, the growth rate of the ITS decreased for the same reason as the UCS.

### 3.5. Freeze–Thaw Cycle Test Results

#### 3.5.1. Qualitative Changes

**Figure 9:**
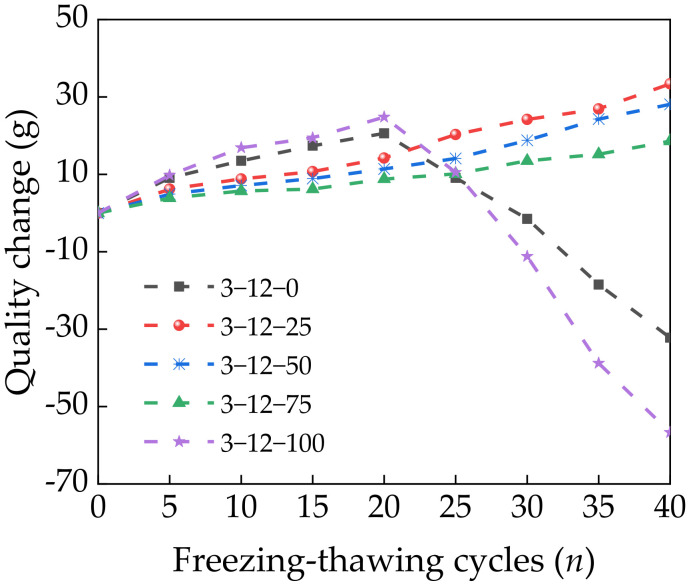
Changes in specimen quality with freeze–thaw cycles.

Figure 9 shows that the specimen quality increased with the number of freeze–thaw cycles up to 20 cycles. However, saturation by immersion caused a large amount of liquid water to fill the microcracks of the specimens. During the freezing process, the liquid water became solid ice, which resulted in volume expansion and a frost heaving force. This generated and expanded new microcracks in the interfacial transition zone of the pore structure [35]. During the thawing process, water filled the newly generated microcracks, which increased the water content of the specimens. The combined effects of the increased water content and surface aggregate spalling initially increased the specimen quality. At 25 freeze–thaw cycles, the surface of the specimens with 0% and 100% SS spalled, and the specimen quality began to decline. However, the quality of the specimens with 25%, 50%, and 75% SS continued to increase with further freeze–thaw cycles. This is because including a suitable amount of SS increased the amounts of C–S–H and other gel substances generated by the reactions in Equation (1). These gels wrapped the surfaces of the fly ash and CRM particles, which increased the density of the interfacial transition zone. This improved the stability and compactness of the specimens and limited the generation and expansion of microcracks. At 100% SS, the rough surface of the SS contained more pores, so the internal compactness of the mixture was low. For the first few freeze–thaw cycles, a large amount of water migrated inside the specimens, which increased the mass. With more freeze–thaw cycles, the repeated freezing and thawing of the pore water produced additional stress on the pore walls. This cycle generated and expanded microcracks at the interface between the cement and aggregate, which caused the surface of the specimen to spall and reduced the mass of the specimen.

#### 3.5.2. Damage Analysis of the Unconfined Compressive Strength

**Figure 10:**
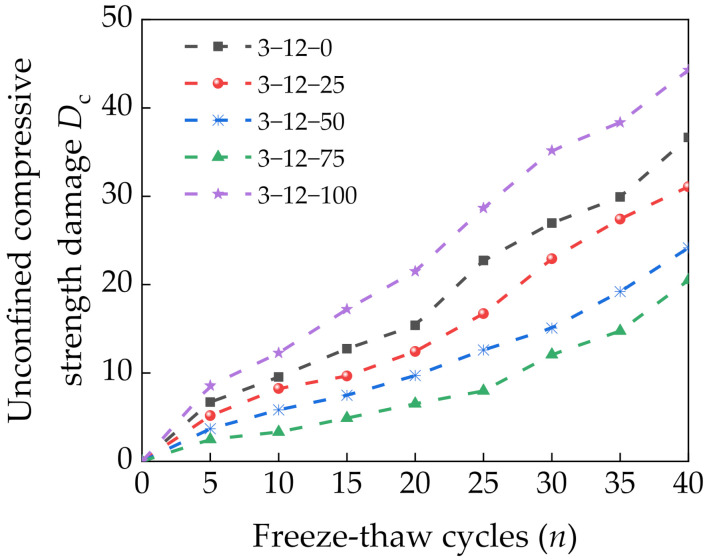
Relationship between freeze–thaw cycles and the unconfined compressive strength damage *D*_c_.

The UCS damage *D*_c_ of a specimen is defined as follows [36]:

(2) $$D_{c}=1-\frac{P_{n}}{P_{0}}$$

where *P*_n_ is the UCS of a specimen for the *n*th freeze–thaw cycle (MPa) and *P*_0_ is the UCS of the specimen before freezing and thawing (MPa). Figure 10 shows the variation in *D*_c_ of specimens with the number of freeze–thaw cycles. Increasing the number of freeze–thaw cycles gradually increased *D*_c_. At 40 freeze–thaw cycles, the specimens with 0% and 100% SS had *D*_c_ of 42.7% and 53.2%, respectively. These results indicate significantly higher damage than for the specimens with 25–75% SS. Increasing the SS content initially decreased and then increased *D*_c_. This shows that incorporating an appropriate amount of SS is conducive to improving the frost resistance of a mixture. This is because SS can react with cement and fly ash to form products that fill up the pores and intertwine in the mixture, which increases the compactness of the specimen. At 75% SS, the appropriate amount and uniform distribution of spherical bubbles offset part of the seepage pressure, which effectively hindered water intrusion and reduced the damage from the frost heaving force and seepage pressure on the specimen [37,38]. Increasing the SS content further resulted in an excessive amount of spherical bubbles that accelerated the connection and formation of pores. This promoted the intrusion of water, increased the frost heaving force caused by water condensation into ice, and reduced the UCS of the specimen.

#### 3.5.3. Strength Attenuation Model Based on Ultrasonic Testing

Under the action of freeze–thaw cycles, the damage accumulated by the specimens inevitably led to the development of cracks. The internal cracks of the specimens absorb and dissipate the energy of propagating acoustic waves. According to the working principle that ultrasonic rays bypass defects and propagate along the minimum energy path, ultrasonic waves can be used to detect defects inside specimen. When an ultrasonic wave passes through a defect, it attenuates, which decreases the wave velocity [39]. Therefore, ultrasonic technology could be used to detect the damage state of specimens subjected to freeze–thaw cycles.

**Figure 11:**
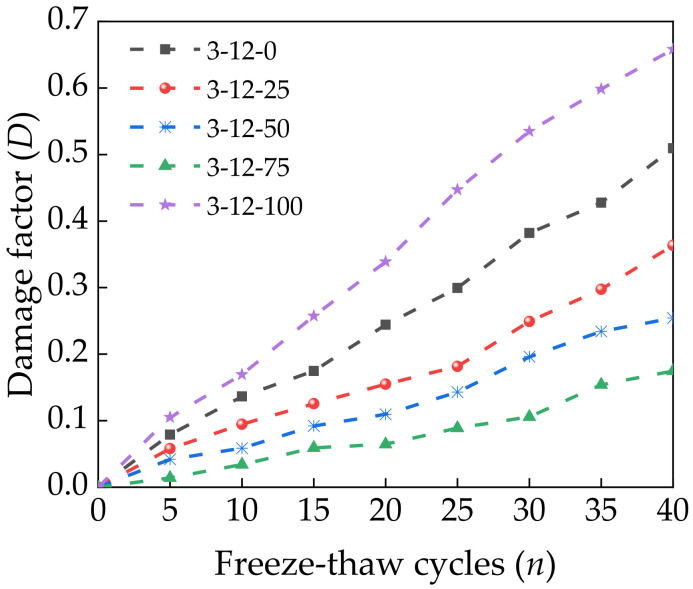
Relationship between freeze–thaw cycles and the freeze–thaw damage factor *D*.

Increasing the number of freeze–thaw cycles caused the cracks inside the specimens to grow rapidly and expand. This changed the path of ultrasonic waves passing through the specimens, which was directly reflected by changes in the ultrasonic time and wave velocity. The relative dynamic elastic modulus *E*_r_ is generally used to represent the degree of damage caused by freeze–thaw cycles [40,41,42]. The damage factor *D* can also be used to evaluate damage and has an initial value of 0. *E*_r_ and *D* can be calculated as follows:

(3) $$E_{r}(n)=\frac{E_{n}}{E_{0}}=\frac{V_{n}^{2}}{V_{0}^{2}}=\frac{{\left(l/t_{n}\right)}^{2}}{{\left(l/t_{0}\right)}^{2}}=\frac{t_{0}^{2}}{t_{n}^{2}}$$

(4) $$D=1-E_{r}(n{)}^{\;}$$

where *E*_n_ is the dynamic elastic modulus of a specimen after the *n*th freeze–thaw cycle (MPa) and *E*_0_ is the initial dynamic elastic modulus of the specimen (MPa). *V*_n_ is the longitudinal wave velocity after the *n*th freeze–thaw cycle (km/s), and *V*_0_ is the initial longitudinal wave velocity of the specimen (km/s). *l* is the initial length of the specimen (mm). *t*_n_ is the sound time of the specimen after the *n*th freeze–thaw cycle, and *t*_0_ is the initial sound time of the specimen. Ultrasonic testing was performed on specimens after standard curing for 28 days, and Figure 11 shows the calculated relationship between *D* and the number of freeze–thaw cycles according to Equations (3) and (4).

**Figure 12:**
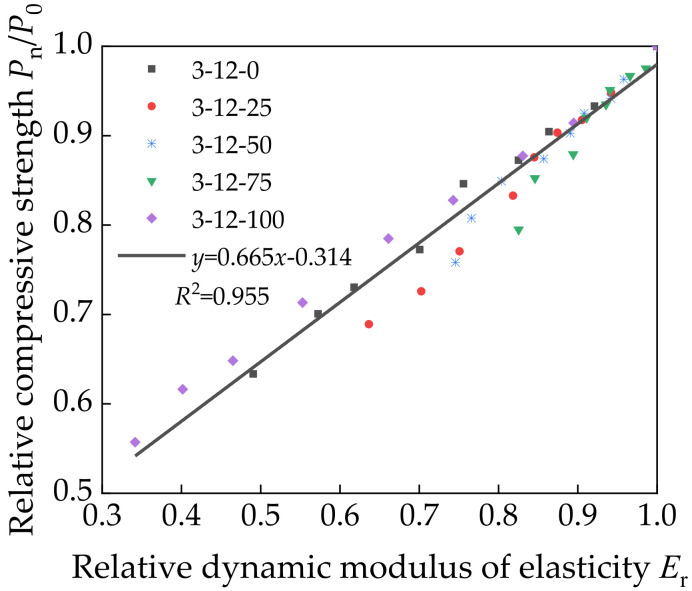
Relationship between the relative dynamic modulus of elasticity and relative compressive strength.

*D* had a similar growth law as *D*_c_. The relationship between *E*_r_ and the relative compressive strength of a specimen after the freeze–thaw cycle test has been shown to be approximately linear [43]. Therefore, the relationship between *E*_r_ and the relative compressive strength of a specimen can be expressed as follows:

(5) $$\frac{P_{n}}{P_{0}}=aE_{r}^{}+b$$

where the values of *a* and *b* are obtained by linear regression analysis. Figure 12 shows the curve fitted to the relationship between *E*_r_ and the relative compressive strength.

The fitting analysis resulted in the following equation:

(6) y=0.665x−0.314

Figure 12 shows that Equation (6) is in good agreement with the experimentally calculated value with *R*^2^ close to 1. This indicates a highly linear relationship between *E*_r_ and the relative compressive strength of a specimen. The linear equation has high accuracy, which can be used for strength detection by ultrasonic field on the premise of ensuring the construction quality. It has the advantages of time saving and labor saving. However, the prediction is established on the basis of a large number of experimental data, which requires high accuracy of ultrasonic equipment in different projects.

### 3.6. Dry Shrinkage Test Results

**Figure 13:**
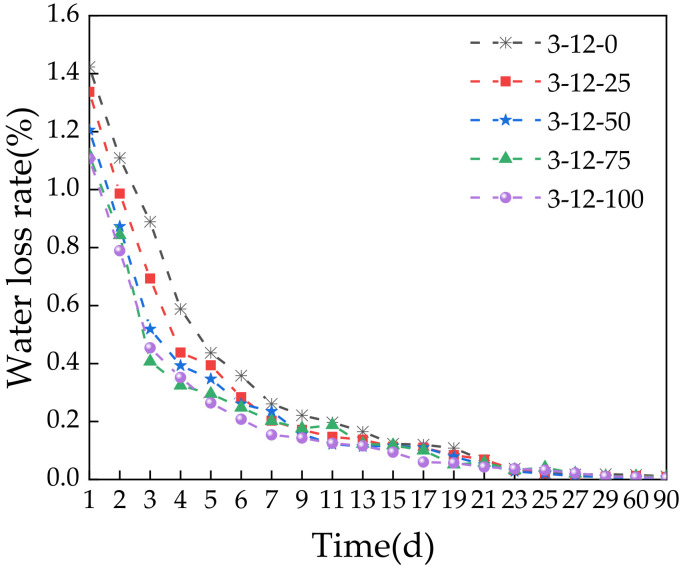
Relationship between the storage time and water loss rate.

The dry shrinkage test was used to obtain the relationships between the curing time of the specimens and the water loss rate, dry shrinkage strain, and dry shrinkage coefficient. Figure 13 shows that the water loss rate of the specimens decreased with increasing curing time regardless of the SS content. At 0–7 days, the specimens showed a clear decrease in the water loss rate, especially for the first 3 days. After 7 days, the decrease in the water loss rate slowed down. This indicates that the water loss of the specimens mainly occurred in the first 7 days. This is because the hydration rate was high when specimens were saturated, which consumed large amounts of water. In addition, evaporation further accelerated the early dehydration. Over time, the water content in the specimens decreases. The hydration of cement and SS slowed down because the generated products wrapped the surfaces of the cement and SS particles. As the reaction rate slowed down, the water consumption decreased, which slowed down the water loss rate. Increasing the SS content reduced the water loss rate of the specimens. This is because the SS content affected the moisture content. Increasing the SS content reduced the moisture content and thus the amount of water available. Thus, less water was consumed over the same amount of time compared with the specimens with no or little SS content. In addition, less evaporation took place. These results indicate that early curing and wetting measures should be strengthened for actual construction so that the base material can quickly improve in strength and to prevent excessive water evaporation to reduce cracking caused by early shrinkage.

**Figure 14:**
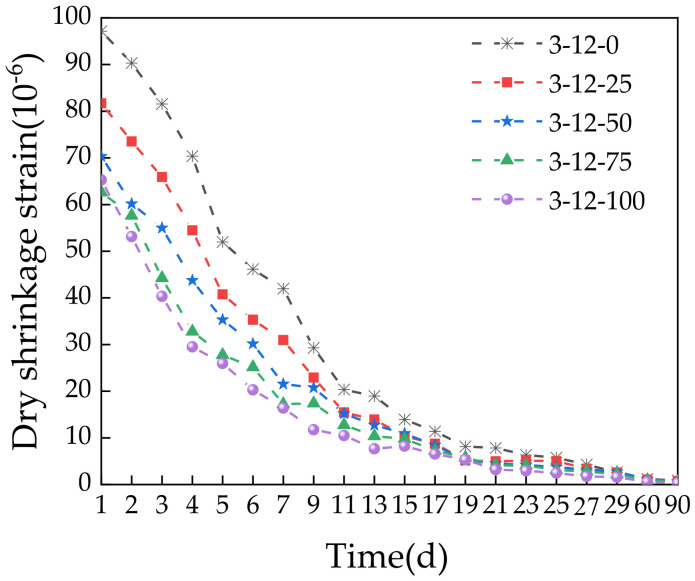
Relationship between the time and dry shrinkage strain.

Figure 14 shows that the dry shrinkage strain of the specimens gradually decreased with increasing time. The rate of decrease was initially fast and then flattened at later stages, which is similar to the water loss rate. This is because the dry shrinkage strain is closely related to the water loss rate, and the dry shrinkage of a mixture is largely caused by water loss. A higher water loss rate reduces the distance between internal particles, which increases the dry shrinkage strain [44]. The dry shrinkage strain was decreased with increasing SS content. This indicates that adding SS can mitigate drying shrinkage to some extent. This is because SS can expand to some degree. The f-CaO and periclase in SS react with water to form Ca(OH)_2_ and Mg(OH)_2_, which increases the volume and compensates for part of the drying shrinkage. Thus, SS can be used to control the cracking of road bases due to dry shrinkage to some extent.

**Figure 15:**
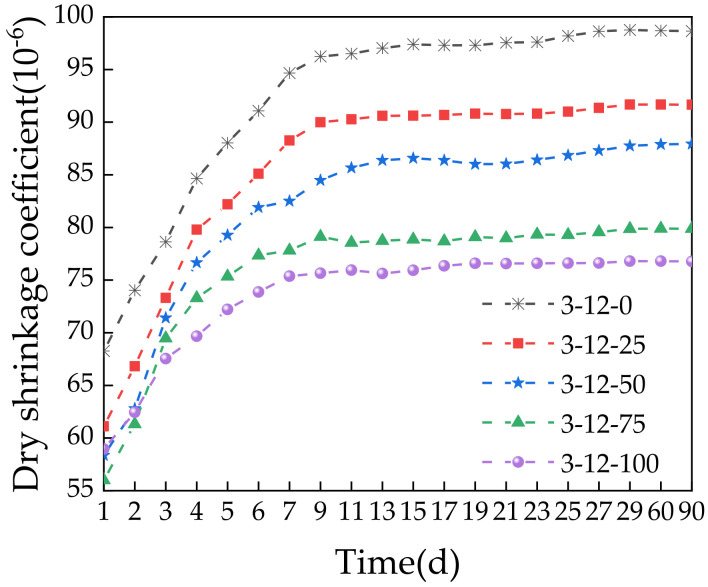
Relationship between the time and dry shrinkage coefficient.

Figure 15 shows that the dry shrinkage coefficient of the specimens increased with time. The growth rate was initially fast and then flattened at later stages. The dry shrinkage coefficient was largest for the specimen with 0% SS at 98.76 × 10^−6^. Increasing the SS content to 25%, 50%, 75%, and 100% reduced the dry shrinkage coefficient by 7.2%, 11.0%, 19.1%, and 22.2%, respectively. This indicates that incorporating SS significantly reduced the shrinkage. This is because the capillary shrinkage caused by the evaporation of water leads to dry shrinkage deformation of the mixture. However, the volume expansion from SS reacting with water compensates for this defect, which effectively alleviates the drying shrinkage. Thus, adding SS can reduce the growth of cracks in pavement [45]. For actual construction, adding an appropriate amount of SS will not only increase the strength but also effectively improve the shrinkage performance of the road base.

### 3.7. Temperature Shrinkage Test Results

**Figure 16:**
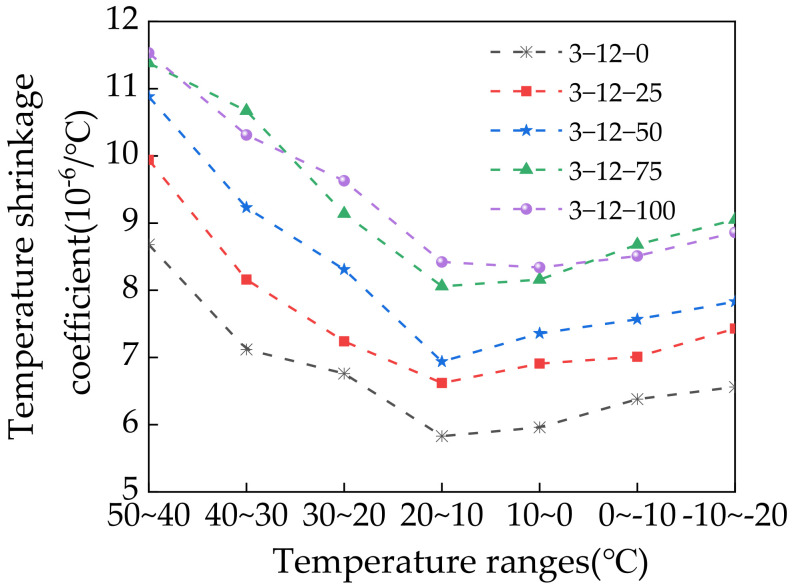
Temperature shrinkage coefficient in different temperature ranges.

The temperature shrinkage coefficient can be calculated as follows:

(7) $$\alpha_{t}=\frac{\varepsilon_{i}}{t_{i}-t_{i-1}}+\beta_{s}$$

where *α*_t_ is the temperature shrinkage coefficient (10^−6^/°C), *t*_i_ is the *i*th temperature range (°C), *ε*_i_ is the shrinkage strain at temperature *i*, and *β*_s_ is the thermal compensation coefficient (10^−6^/°C). Table 5 presents the temperature shrinkage test results of the specimens according to the SS content. Figure 16 shows the temperature shrinkage coefficient curves at different temperature ranges.

**Table 5:** Results of the temperature shrinkage test.

Specimen Number Temperature (°C)	Temperature Shrinkage Coefficient (10^−6^/°C)				
	3-12-0	3-12-25	3-12-50	3-12-75	3-12-100
50–40	8.68	9.94	10.88	11.38	11.53
40–30	7.12	8.16	9.23	10.67	10.31
30–20	6.76	7.24	8.31	9.14	9.63
20–10	5.83	6.62	6.94	8.06	8.42
10–0	5.96	6.91	7.36	8.16	8.34
0 to −10	6.38	7.01	7.57	8.68	8.51
−10 to −20	6.56	7.43	7.83	9.05	8.86
Average temperature shrinkage coefficient (10^−6^/°C)	6.76	7.62	8.30	9.31	9.37

Note: 3-12-X refers to the mixture compositions: 3% cement, 12% fly ash, and X% steel slag.

The temperature shrinkage coefficient increased with increasing SS content, which indicates that incorporating SS increased the temperature shrinkage strain of the specimens. At a temperature range of 50 °C–40 °C, 100% SS resulted in a maximum temperature shrinkage coefficient of 11.53, which was 32.8% higher than that without SS. Decreasing the temperature initially decreased and then increased the temperature shrinkage coefficient of the specimens, with the minimum value at 20 °C–10 °C. The temperature shrinkage coefficient of the specimens varied at different temperature ranges. When the temperature was decreased from 50 °C to 10 °C, the temperature shrinkage coefficient decreased at a faster rate. When the temperature was decreased from 10 °C to −20 °C, the temperature shrinkage coefficient gradually increased but at a slow rate. Adding SS increased the temperature shrinkage coefficient because SS reacts with water to generate hydration products and secondary minerals. Compared with CRM, it is more sensitive to changes in temperature. Note that, in addition to the material properties, many other factors affect the temperature shrinkage strain such as the cement type, mixture gradation type, mixing method, and measurement method [46].

## 4. Strength Formation Mechanism

**Figure 17:**
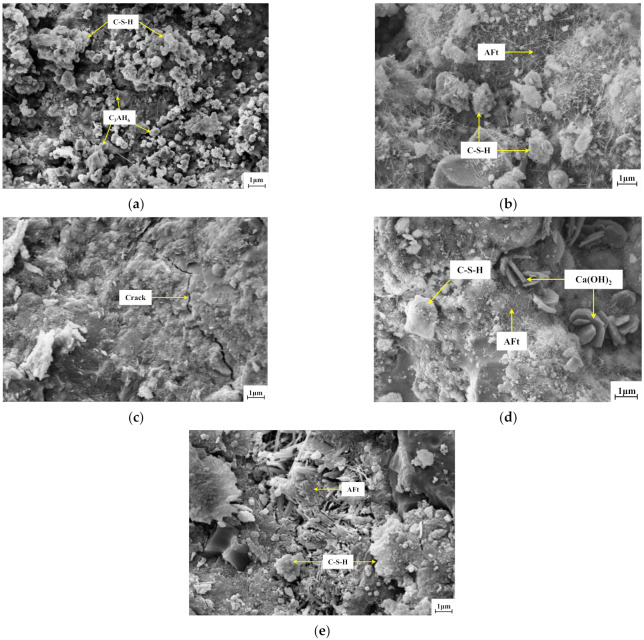
Microstructures of specimens according to the SS content and age: (**a**) 0% SS 7 days; (**b**) 75% SS 7 days; (**c**) 100% SS 7d ays; (**d**) 75% SS 28 days; and (**e**) 75% SS 90 days.

In addition to the mechanical compaction effect, adding SS to the mixtures gradually reduced the pores between the SS and CRM. This made the internal structure more compact and improved the strength. The active substances such as cement, fly ash, and SS reacted continuously over time to generate various hydration products. This gradually formed a network of connections that made the mixture more compact and continuously improved the strength [47]. SEM was used to observe the strength formation and development of mixtures according to the SS content and curing time. Figure 17a–c show the microstructures of specimens with 0%, 75%, and 100% SS, respectively, at 7 days; Figure 17d,e show the microstructures of the specimen with 75% SS at 28 and 90 days, respectively.

At the microscopic scale, the strength formation of the SS–CRM mixture stabilized with cement and fly ash can be characterized as hydration products moving from an amorphous gel to low crystallinity and finally to high crystallinity. Figure 17a shows a large amount of octahedral C_3_AH_6_ dispersed in the mixture without SS, and a small amount of C–S–H gel polymerized together for strength formation. Figure 17b shows that 75% SS increased the active substances, which promoted the hydration of cement, generated more C–S–H gels and rod-like Aft. This gradually formed a spatial network structure, but there were obvious pores, and the distribution was relatively loose. Figure 17c shows that 100% SS changed the morphology of the hydration products from a messy and loose honeycomb structure to a compact and uniform structure. However, obvious cracks were observed, which decreased the strength. Figure 17d shows the intersection between reticular C–S–H gel and needle-like ettringite Aft crystal, which gradually connected to the whole. Compared with the structure at 7 days, the pores decreased, and the density increased. Meanwhile, the Ca^2+^ concentration in the liquid phase increased, which resulted in the crystallization of Ca(OH)_2_ and filling of the pores. Figure 17e shows that the quantity and morphology of hydration products changed significantly with the curing time. Large volumes of hydration products were accumulated by overlapping and stacking, which increased the overall density of the structure and the strength of the material at the macroscale.

Based on the test results and observations of the microstructure, the strength formation mechanism of the mixtures can be explained as follows. First, cement, fly ash, and fine aggregate fill the spaces between the SS and CRM particles to form a microaggregate effect, which improves the compactness and stability of the cement mortar interface [48]. Second, fly ash and SS have high activity and contain alkaline oxides that fully react with the cement hydration product Ca(OH)_2_ over time to form a dense C–S–H gel and ettringite. The formation of the C–S–H gel binds the fly ash and SS particles with the CRM particles more closely, which improves the overall strength and compactness [49].

## 5. Economic Benefit Analysis

In recent years, the state has increased efforts to protect the environment. Most of the quarries around Yinchuan have been closed and rectified, which has made the construction of highway engineering in Yinchuan enter a bottleneck period. At the same time, the prices of stone and cement have surged, and the price of stone has exceeded 60 yuan/t, and the price of cement has exceeded 450 yuan/t. However, a large amount of local steel slag is accumulated and the sales are very limited, which seriously affects the lives of local residents. Under the pressure of the environmental protection department and the surrounding citizens, an effective treatment is urgently needed.

According to the research results of the road performance of cement fly ash stabilized steel slag-concrete recycled aggregate mixture, with steel slag content of 75%, concrete recycled aggregate content of 25%, cement content of 3% and fly ash content of 12%, combined with the local market raw material prices in Yinchuan City, the cost of unit area under a certain thickness is calculated, and compared with the cost of ordinary semi-rigid base cement stabilized macadam. The width of the lane is 3.5 m two-way four lanes, and the thickness is 0.3 m. The material cost of the two mixtures is calculated. The economic analysis is shown in Table 6.

As can be seen from the table, the same paved one kilometer of the base, the use of cement fly ash stabilized gravel material costs 766,328.8 yuan, while the use of cement fly ash stabilized steel slag-concrete recycled gravel material costs only 568,905.0 yuan, only consider raw materials, per km can save nearly 200,000 yuan. Thus, using cement fly ash stabilized steel slag-concrete recycled gravel has good economic benefits.

## 6. Conclusions

The physical and chemical properties of the raw materials were used to evaluate the mechanical properties, frost resistance, and crack resistance of five SS–CRM mixtures stabilized with cement and fly ash according to the SS content. Experimental results combined with microstructural analysis and ultrasonic detection obtained the following main conclusions:

- The Immersion expansion rate test showed that the mixture mixed with steel slag had no damage in the expansion process and had good volume stability. The higher the steel slag content, the greater the maximum dry density of the mixture, and the smaller the optimum moisture content; the unconfined compressive strength and indirect tensile strength increased first and then decreased with the increase of steel slag content, and reached the maximum when the content was 75%. The compressive strength at 7 days, 28 days, 56 days and 90 days was 5.32 MPa, 9.78 MPa, 10.52 MPa and 11.02 MPa, respectively, which meets the strength requirements of highways and first-class highways in China under extremely heavy and special traffic grades.
- Under the action of freeze-thaw cycle, the mass change and strength damage of the mixture with 25%, 50% and 75% steel slag content decreased, and the frost resistance was improved. The damage of unconfined compressive strength after freeze-thaw cycles was detected by ultrasonic testing technology, and the strength attenuation model between relative dynamic elastic modulus and relative compressive strength was proposed to guide the construction in cold regions.
- With the increase of steel slag content, the dry shrinkage coefficient of the mixture decreases and the temperature shrinkage coefficient increases. The dry shrinkage coefficients of the mixture with 25%, 50%, 75% and 100% steel slag are 7.2%, 11.0%, 19.1% and 22.2% lower than those without steel slag, respectively. The incorporation of steel slag helps to reduce the occurrence of dry cracks. With the decrease of temperature, the temperature shrinkage coefficient of the mixture first decreases and then increases. In order to reduce the occurrence of cracks in the later stage, it is suggested that the construction temperature should be between 5 °C and 25 °C in the construction process of cement fly ash stabilized steel slag-concrete recycled aggregate base.
- At present, the related research focuses on the macroscopic properties of steel slag mixture. This study reveals the strength formation mechanism of cement fly ash stabilized steel slag-concrete recycled aggregate mixture from the microscopic aspect; that is, the incorporation of steel slag increases the amount of cementitious materials in the mixture and distributes them inside the pores. The hardened slurry structure is denser and the road performance of the mixture is improved. It provides a theoretical basis and reference for the application of steel slag in road base, and effectively realizes the efficient recycling of industrial solid waste.

## Figures and Tables

**Table 1:** Chemical composition of materials (%).

Materials	Al_2_O_3_	SiO_2_	CaO	Fe_2_O_3_	Na_2_O	MgO	K_2_O	FeO
Cement	5.62	22.41	62.78	3.56	0.23	1.28	0.72	—
Fly ash	28.65	45.38	7.25	5.47	0.73	1.25	2.08	—
Steel slag	3.16	11.84	41.28	18.23	0.12	6.13	0.08	8.75

**Table 2:** Technical indices of the cement.

Index	Fineness (%)	Stability (mm)	Setting Time (min)		Compressive Strength (MPa)		Flexural Strength (MPa)	
			Initial Setting	Final Setting	3 days	28 days	3 days	28 days
Measured Value	3.56	2.14	198	453	15.36	39.66	3.85	8.24
Normative Value	≤10.0	≤5.0	≥45	≤600	≥11.0	≥32.5	≥2.5	≥5.5

**Table 3:** Physical properties of materials representing performance indices.

Index	Apparent Density (g·cm^−3^)	Water Absorption (%)	Crushing Value (%)	Los Angeles Wear Value (%)	Needle-Like Content (%)	Immersion Expansion Rate (%)	Autoclave Pulverization Rate (%)
SS	3.158	1.78	16.3	12.3	2.3	1.45	4.35
CRM	2.514	2.14	22.6	18.8	6.2	-	-

SS: steel slag, CRM: concrete-recycled macadam.

**Table 4:** Mix design.

SS:CRM	SS (mm)				CRM (mm)			
	0–4.75	4.75–9.5	9.5–19	19–26.5	0–4.75	4.75–9.5	9.5–19	19–26.5
A (0%:100%)	-	-	-	-	32%	24%	28%	16%
B (25%:75%)	8%	6%	7%	4%	24%	18%	21%	12%
C (50%:50%)	16%	12%	14%	8%	16%	12%	14%	8%
D (75%:25%)	24%	18%	21%	12%	8%	6%	7%	4%
E (100%:0%)	32%	24%	28%	16%	-	-	-	-

SS: steel slag, CRM: concrete-recycled macadam.

**Table 6:** Economic analysis of two mixtures.

Mixture Type	Macadam (Yuan/t)	CRM (Yuan/t)	SS (Yuan/t)	Cement (Yuan/t)	Fly Ash (Yuan/t)	Material Cost (Yuan/km)
Cement fly-ash stabilized macadam	60	-	-	450	20	766,328.8
Cement fly-ash stabilized steel slag-oncrete recycled macadam.	-	35	25	450	20	568,905.0

## Data Availability

The data presented in this study are available on request from the corresponding author.
